# The Potential of South African Herbal Tisanes, Rooibos and Honeybush in the Management of Type 2 Diabetes Mellitus

**DOI:** 10.3390/molecules23123207

**Published:** 2018-12-05

**Authors:** Olawale R. Ajuwon, Ademola O. Ayeleso, Gbenga A. Adefolaju

**Affiliations:** 1 Redox Biology Research Unit, Department of Biochemistry, Federal University, Oye-Ekiti 371010, Nigeria; 2 Department of Biochemistry, Adeleke University, Ede, Osun State 232101, Nigeria; ademola.ayeleso@adelekeuniversity.edu.ng; 3 Department of Pre-Clinical Sciences, University of Limpopo, Sovenga 0727, South Africa; gbenga.adefolaju@ul.ac.za

**Keywords:** tea, rooibos, honeybush, polyphenol, type 2 diabetes

## Abstract

Diabetes mellitus is a metabolic disease that can lead to high morbidity, mortality and long-term complications. Available treatment strategies, which are mainly based on treating hyperglycemia, with insulin and other pharmacological agents are not completely efficient and can even lead to development of unwanted side effects. Scientific evidence suggests that bioactive compounds from teas and other plant-based foods, which are known source of natural antioxidants, could be an attractive strategy to preferentially treat and manage type 2 diabetes mellitus (T2DM) and thus, have significant therapeutic implications. In this review, we attempt an in-depth analysis and discussion of the current progress in our understanding of the antidiabetic potential of two commercialized South Africa herbal tisanes—Rooibos and Honeybush and their polyphenols.

## 1. Introduction

Diabetes mellitus is one of the most common global diseases that cause substantial morbidity, mortality and long-term complications including retinopathy, nephropathy, peripheral nerve damage and cardiovascular disease. According to the International Diabetes Federation, diabetes currently affects an estimated 429 million people worldwide and with the prevalence increasing at an alarming rate, the figure is projected to increase to around 625 million people by 2045 [1]. Diabetes mellitus is broadly classified as: (i) insulin-dependent diabetes mellitus (IDDM) or type 1 diabetes mellitus (T1DM) which account for about 5 to 10% of diabetes and is characterized by inability to produce insulin because of autoimmune destruction of pancreatic β cells [2]; and (ii) non-insulin-dependent diabetes mellitus (NIDDM) or type 2 diabetes mellitus (T2DM). T2DM is characterized by a dysfunctional carbohydrate, lipid and protein metabolism resulting from a progressively impaired insulin secretion and/or insulin resistance. At the initial stage of the disease, insulin resistance is compensated by increased pancreatic β-cells mass and insulin secretion, however, as the disease progresses, pancreatic β-cells mass and function gradually decline leading to decreased uptake of glucose into skeletal muscle, liver and adipose tissue resulting in uncontrollable hyperglycemia [3]. Several behavioral, lifestyle and biological risk factors are known to play significant role in the development and progression of T2DM including obesity, sedentary life style, older age, high blood pressure, unfavorable lipid profile, cigarette smoking and genetic pre-disposition [4].

Experimental and clinical evidence has shown that oxidative stress, endothelial dysfunction and chronic inflammation are interrelated in the pathophysiology of T2DM. Overproduction of free radicals has been suggested to be involved in the onset of T2DM and diabetic complications with oxidative stress proposed as a pathogenic mechanism linking insulin resistance with β-cells and endothelial dysfunction, impaired glucose tolerance and overt diabetes [5]. Hyperglycemia is reported to lead to overproduction of reactive oxygen species (ROS) which may result in β-cells dysfunction due to decrease in β-cells mass attributed to oxidative stress-induced apoptosis [6]. Impairment of insulin-stimulated glucose uptake by fat and muscle during diabetes causes blood levels of glucose concentrations to remain high. As a result, glucose uptake by insulin-independent tissues increases. The increased glucose flux enhances oxidant production and impairs antioxidant defenses via several interacting pathways including, hyperglycemia-induced mitochondrial dysfunction, increased polyol pathway flux, activation of protein kinase C, increased production of advanced glycation end-products (AGE), increased hexosamine pathway flux, as wells as endoplasmic reticulum stress [7,8]. There are reports linking ROS to induction of insulin resistance [9,10], impairment of insulin synthesis [11] and β-cell insulin secretion [12]. Additional evidence supporting ROS contribution to the onset, progression and pathological consequences of T2DM and diabetic complications comes from reports that biomarkers of oxidative damage including malondialdehyde, F2-isoprostane, protein carbonyls and advanced glycation end-products (AGE) are reportedly raised in either the plasma, erythrocytes or liver of streptozotocin (STZ)-induced diabetic rats [13,14,15,16] and in the plasma or urine of diabetic patients [17,18,19]. Additionally, levels of cellular non-enzymatic antioxidants such as vitamin E, vitamin C and reduced GSH are reduced in different tissues of experimentally induced diabetes [20,21] and in diabetic patients [19,22,23,24]. Another evidence though indirect, that supports the role of oxidative stress in diabetes and diabetic complications is the modulation of the antioxidant enzymes network but this effect is considered to be highly variable and depends on the model of diabetes used and the type of tissue evaluated [2]. Hyperglycemia-induced oxidative stress also induces endothelial dysfunction, increase pro-inflammatory and pro-coagulant factors expression, induce apoptosis and impair nitric oxide release, all factors that play a central role in the pathogenesis of micro- and macro-vascular diabetic complications [8].

The available treatment modality for diabetes is essentially the treatment of hyperglycemia and this relied on insulin and other pharmacological agents such as sulfonylureas and metformin. These treatment strategies are not completely efficient as indicated by rise in morbidity and mortality rate in diabetic patients [25]. The pharmacological agents are also reported to cause various side effects, such as weight gain, hypoglycemia, fluid retention and heart failure limiting their use [26]. Evidence has indicated that weight loss, as well as life style and behavioral changes are effective in the management of diabetes [27], however patients’ compliance and acceptability are poor. On the backdrop of these drawbacks and the fact that about 80% of people affected by T2DM lives in low- and middle-income countries where accessibility to adequate medical care may not be available, it is therefore essential to develop new antidiabetic agents which can control hyperglycemia and with fewer side effects.

The involvement of hyperglycemia-induced oxidative stress in the pathophysiology of T2DM suggests that bioactive compounds from teas and other plant derived foods, which are known source of natural antioxidants could be an attractive strategy to preferentially manage T2DM and thus have significant therapeutic implications. The concept that the consumption of tea might reduce the risk and prevalence of T2DM is strongly supported by direct research findings [28]. Evidence from in vitro studies [29,30,31], supported by in vivo [32,33,34,35,36,37,38] and various epidemiological and human interventional clinical trials [39,40,41,42,43,44,45,46,47] have shown that *Camelia sinensis* based teas possess unquestionable antidiabetic properties.

Rooibos (*Aspalathus linearis*) and honeybush (*Cyclopia* species) are two South African tisanes that are currently enjoying an appreciable degree of commercial success globally. Both herbal teas has a flavonoid profile that is distinctly different from those found in *Camelia sinensis*. Due to their rich content of different compounds with antioxidant and other health properties, both herbal teas are gaining more attention worldwide because of their potential for clinical use. Different biological properties have been reported for both herbal teas (Reviewed by Marnewick J.L. in Reference [48]). Therefore, in this review, we present a comprehensive analysis of evidence available on the potential anti-diabetic benefits that may be associated with the consumption of two South African herbal tisanes, (*Aspalathus linearis* and *Cyclopia* species) and some of their natural bioactive compounds.

## 2. South African Herbal Tisanes

Next to water, tea is the most consumed non-alcoholic beverage in the world. Teas are classified as traditional (from *Camellia sinensis*) or herbal (non-*Camellia sinensis*) teas or tisanes. Herbal teas are prepared by means of infusion, decoction or maceration of seeds, fruits, flowers, leaves, stems and roots from different plants and herbs. They are becoming increasingly popular all over the world in recent years due to their fragrance, aroma and health promoting effects. The African continent has a rich diversity of plants, with evidence suggesting that 25% of total number of higher plants in the world (estimated to be over 30,000 species) is found in Africa, south of the Sahara [49,50]. Because of the amazing floral and cultural diversity of South Africa, many plant species are used traditionally for medicinal purposes. Van Wyk and Gericke [51] reported that in South Africa as a whole, an estimated 3000 plants species are used as medicines. Despite this large number of plant species being used as medicines, only very limited number has been commercialized, although, as many as about 500 are traded in large quantities in informal markets [50]. Herbal teas endemic to South Africa are some of the medicinal plants that are currently enjoying various degree of commercial success. Rooibos (*Aspalathus linearis*) and honeybush (*Cyclopia* species) are very popular even outside South Africa because scientific evidence has shown that they possess various medicinal and therapeutic properties including antioxidant, anti-inflammatory, anticancer and chemo-preventive effects [48].

### 2.1. Rooibos Herbal Tea (Aspalathus linearis) (Family Fabaceae; Tribe Crotalarieae)

Rooibos herbal tea is made from stem and leaves of *Aspalathus linearis*, a shrubby plant with bright green needle-shaped leaves and small yellow flowers, which can grow up to 2 m in height. The rooibos plant is indigenous to South Africa, specifically, the Cederberg mountains region around Clamwilliam, North of Cape Town. Consumed in its traditional fermented (red) or unfermented (green) form, rooibos herbal tea is naturally caffeine-free and has low level of tannin (about 3.2 to 4.4%) [52]. Rooibos differ from *Camellia sinensis* based teas in its polyphenolic composition (See Table 1). Rooibos contain two distinctive monomeric flavonoids; aspalathin, a C-C linked dihydrochalcone glucoside [53] and aspalalinin, a cyclic dihydrochalcone [54] which to date are only isolated in rooibos. Other polyphenolic constituents identified in rooibos includes the 3-dehydroxy dihydrochalcone glucoside, nothofagin [55], flavones including orientin, iso-orietin, vitexin and iso-vitexin, luteolin, luteolin-7-*O*-glucoside and chrysoeriol, flavanones such as dihydro-orietin, dihydro-isoorietin and hemiphlorin, as well as flavanols, including quercetin, quercetin-3-roninobioside, iso-quercitrin, chrysoeriol and rutin [48,56,57,58,59,60]. Other phenolic compounds that have been isolated from rooibos are phenolic acids, lignans, (+)-catechin, a phenylpyruvic acid glycoside (PPAG), coumarins, esculentin and esculin [54,55,56]. The fermentation process gives rooibos its unique reddish brown color, taste and aroma. The process is also known to decrease the flavonoid content of rooibos, as the herbal teas prepared from the unfermented (green) rooibos are reported to have higher antioxidant capacity compared to those prepared from the traditional fermented rooibos [48]. The rich content of polyphenolic bioactive constituents found in rooibos has increased its popularity all over the world because of its potential for clinical use.

The biological activity of rooibos and its polyphenols has been reported in in vitro, in vivo and in a few human studies. The ability of rooibos to modulate oxidative stress by inhibiting lipid peroxidation and augment glutathione redox status has been reported in rat sperm and liver [61,62,63] and in humans at the risk of cardiovascular disease [64]. Furthermore, anti-inflammatory [63,65,66] and chemopreventive [67], as well as cardio-protective [68], effects have all been reported for rooibos and some of its polyphenolic constituents.

#### Antidiabetic Effects of Rooibos Herbal Tea and Its Polyphenols

The involvement of oxidative stress in the pathophysiology of diabetes and diabetic complications suggested that natural products with antioxidant and anti-inflammatory properties may be able to exert beneficial effect. Rooibos is a rich source of unique antioxidants which may play important role in the prevention, management and treatment of T2DM. For example, an aqueous and alkaline extract of fermented rooibos when administered to STZ-treated diabetic rats were reported to reduced biochemical markers characterizing hepatotoxic effects, advanced glycation end products and malondialdehyde in the plasma and/or tissues of the rats [69]. The antioxidant potential of fermented rooibos extract in STZ-induced diabetic rat model was further highlighted by Ayeleso et al. [70], where it was reported that fermented rooibos extract was able to suppress lipid peroxidation and enhance the activity of antioxidant enzymes, superoxide dismutase (SOD) and glutathione peroxidase (GPx)in the plasma and liver. Results from an ex vivo study by Dludla et al. [71], showed that aqueous extract of fermented rooibos exhibit cardioprotection on cultured cardiomyocytes derived from diabetic rats subjected to experimentally-induced oxidative stress and ischemia. The molecular mechanisms associated with this protection was reported in a follow-up study by the same group to be due to the ability of aspalathin to protect against high glucose (HG)- and hyperglycemia-induced oxidative damage via the up-regulation of mRNA expression of Nrf2 and its downstream antioxidant targets [72]. Evidence in support of the ability of rooibos and its unique flavonoid, aspalathin to modulate glucose metabolism in the diabetic and non-diabetic conditions abound in literatures. The first evidence of the beneficial effect of aspalathin on glucose metabolism in T2DM was presented in a study by Kawano et al. [73], where aspalathin from fermented rooibos extract was shown to increase glucose uptake and insulin secretion in L6 myotubes and cultured RIN-5F cells respectively. In the same study, dietary aspalathin was reported to suppress elevated fasting blood glucose and alleviate impaired glucose tolerance in db/db diabetic mice.

A similar outcome was reported by Muller et al. [74], where it was showed that an aspalathin-enriched unfermented rooibos extract and pure aspalathin modulates glucose metabolism by inhibiting the activity of α-glucosidase (from *Saccharomyces cerevisae*) and promoting glucose uptake in C2C12 muscle cells while also reducing fasting plasma glucose in STZ-induced diabetic Wistar rats. Consistent with this evidence, Mikami et al. [75] reported that both unfermented rooibos and aspalathin suppressed the elevation of blood glucose that was induced by carbohydrate overload in non-diabetic rats and inhibit the activity of α-amylase and α-glucosidase enzymes (determined as maltase and sucrase from rat intestine), suggesting that this effect is due to the ability of rooibos and aspalathin to inhibit glucose metabolism. Using the same C2C12 muscle cells model, Mazibuko et al. [76], reported that a fermented and an aspalathin-enriched unfermented rooibos extract, both ameliorated palmitate-induced insulin resistance by increasing glucose uptake, mitochondrial activity and ATP production. A more recent study from the same group, using a 3T3-L1 adipocytes model further investigated the molecular mechanisms responsible for the ability of aspalathin-enhanced unfermented extract and pure aspalathin to ameliorate palmitate-induced insulin resistance. Results from the study revealed that both extract and pure aspalathin were able to reverse palmitate-induced insulin resistance as a result of enhanced glucose transporter 4 GLUT4 expression and inhibition of protein kinase C (PKC), NF-κβ and 5′ adenosine monophosphate-activated protein kinase (AMPK) phosphorylation [77]. These findings were corroborated by works which reported an improvement in glucose tolerance in obese (ob/ob) [78] and KK-Ay [79] mice by aspalathin and unfermented rooibos respectively. The two studies also showed that both pure aspalathin and unfermented rooibos enhanced glucose uptake by stimulating AMPK phosphorylation and promoting the translocation of GLUT4 to the plasma membrane in L6 myotubes and pancreatic β-cells.

Atherosclerosis is a major T2DM complications and evidence has shown that vascular inflammation induced by high glucose is key in its initiation and progression. The ability of aspalathin to alleviate vascular inflammation was investigated by Ku et al. [80]. Their results showed that aspalathin suppressed vascular inflammation by inhibiting HG-mediated vascular permeability and expression of cell adhesion molecules in human umbilical vein endothelial cells (HUVEC) and inhibit ROS formation and NF-κβ activation in rats. Furthermore, recent study in diabetic non-human primate supported the antidiabetic potential of aspalathin-enriched unfermented rooibos. Result from the study showed that supplementation of the enriched extract at 90 mg/kg, three times daily with meal, to high-fat fed diabetic Vervet monkeys improves glucose tolerance, protected against LDL oxidation, preserved endogenous coenzyme Q10 levels and decrease oxidative stress [81].

Evidence also abound of the antidiabetic potential of other rooibos polyphenols apart from aspalathin. The dihydrochalcone glucoside, nothofagin was reported to ameliorate hyperglycemia- and LPS-induced vascular inflammation in HUVEC cells and in mice [80,82]. Although studies on the antidiabetic potential of the major flavones present in rooibos are very limited, however, vitexin, orientin and iso-orientin isolated from *Spirodela polyrhiza* were reported to inhibit adipogenesis and decrease C/EBPα and PPARγ protein expression in 3T3-L1 mouse adipocytes [83]. Another study by Ku et al. [84] indicated that orientin was able to inhibit high glucose-induced vascular inflammation in vitro and in vivo. Iso-orientin isolated from *Gentiana olivieri* has been shown to reduce fasting blood glucose, cholesterol and triglyceride level in STZ-induced diabetic rats [85]. Yuan et al. [86] supported this findings showing that iso-orientin ameliorated diabetic complications including lipid toxicity and insulin resistance in high-fructose-fed mice. Vitexin and isovitexin are flavone derivatives of nothofagin. Studies has shown that both vitexin and isovitexin inhibits α-glucosidase activity [87] and in vivo, both compounds are reported to reduce postprandial blood glucose levels when fed orally to sucrose loaded normoglycemic mice [88]. Furthermore, extract rich in vitexin and isovitexin were reported to ameliorate diabetic complications including adipogenesis and AGEs in vitro [89]. Luteolin is present in rooibos in trace amount, although its release from orientin and iso-orientin in the gut may increase the level to appreciable physiological relevance [90]. In diet-induced obese mice, luteolin improved hepatic insulin sensitivity by inhibiting gluconeogenesis [91]. Other studies also indicated that luteolin is able to ameliorate diabetic complications including, protection against diabetes-associated cognitive decline by suppressing oxidative stress [92] and STZ-induced morphological destruction of the kidney [93] in rats.

Quercetin, hyperoside, isoquercitrin and rutin are the major flavanols present in rooibos. There is no literature on the antidiabetic effects of isolated quercetin from rooibos. Rutin modulates glucose metabolism by inhibiting α-glucosidase activity (from *Saccharomyces cerevisae*) and promote glucose uptake in C2C12 muscle cells. In the same study, in STZ-induced diabetes rats, an aspalathin-rutin mixture was reported to reduce blood glucose concentrations over a 6 h monitoring period [74]. Other studies have shown that rutin improved glucose homeostasis by altering glycolytic and gluconeogenic enzymes, decreased postprandial hyperglycemia and slowed down the formation of AGEs in different experimental models [94,95,96].

Phenylpyruvic acid-2-*O*-glucoside (PPAG), first isolated in rooibos by Marais et al. [97], is a major monomeric phenolic acid present in both fermented and unfermented rooibos. Available evidence has shown that PPAG was able to delay the onset of diabetes by preventing cell death and necrosis of pancreatic β-cells from STZ-induced diabetic mice [98]. Furthermore, Muller et al. [99] and Mathijs et al. [100] reported on the hypoglycemic effects of PPAG, where it was shown to reduce fasting blood glucose and insulin levels and improve glucose tolerance and insulin resistance in OBIR rats. The molecular mechanisms of the observed effects, according to these authors was presumably due to the ability of PPAG to increase the expression of glucokinase, GLUT1 and 2, PPARα and suppress cytokine release through inhibition of apoptosis and neogenesis of pancreatic β-cells. A more recent work by Dludla et al. [101] corroborated this earlier observation when they showed that PPAG in combination with metformin attenuates high glucose-induced apoptosis in H9c2 cardiomyocytes. No clinical trials on the effect of rooibos and any of its polyphenolic compounds on diabetic patients were found in the available literature. However, a human study by Francisco [102] revealed that consumption of an aqueous extract of fermented rooibos modulated postprandial glycaemia, lipemia and oxidative stress in healthy volunteers after the intake of a standardized fat meal. Table 2 below show the summary of some studies indicating the anti-type 2 diabetic mellitus potential of rooibos and some of its major polyphenols.

### 2.2. Honeybush Herbal Tea (Cyclopia Vent.)(Family Fabaceae; Tribe Podalyrieae)

Apart from rooibos, honeybush tea is the other South African herbal tea that is currently enjoying some degree of commercial success. Honeybush, like rooibos also belong to the Fynbos biome that account for more than 80% of the plant species in the Cape Flora kingdom. The herbal tea is made from the leaves, stem and flowers of the *Cyclopia* species. The *Cyclopia* species is endemic to South Africa and grow from the Cederberg Mountains north of Citrusdal to the Cape Peninsula in the South and Port Elizabeth in the Eastern Cape region [48,55,103]. There are about 23–24 species of the *Cyclopia* but commercial honeybush herbal tea is made from mainly three species, namely, *Cyclopia intermedia* E. Mey, *Cyclopia subternata* Vogel and *Cyclopia genistoides* (L) Vent [55,103]. *Cyclopia* species are shrubby, woody plants that can grow to between 1.5 m to 3 m in height with trifoliate leaves and pale yellow, sweet smelling flower which gives the herbal tea its characteristic sweet-honey like aroma. The leaves vary in shapes and sizes from species to species, ranging from narrow and pointed at the end to broader and more elongated [103]. Like rooibos, honeybush is caffeine-free and has very low content of tannin compared to *Camellia sinensis* tea, making it a suitable night time beverage and for those experiencing nervousness [48]. The traditional fermented honeybush is the most common honeybush tea on the market, although recently the unfermented green honeybush has been introduced. The fermentation process gives the tea its unique red-brown color and sought-after sweetish flavor.

The phytochemical composition of honeybush is distinctly different from those of rooibos and *Camelia sinensis* (See Table 1). The main polyphenols found in honey bush are xanthones, flavones, isoflavones, flavanones, flavanols and coumestans. De Nysschen et al. [104] who screened methanolic extracts of 22 *Cyclopia* species in 1996, reported that the xanthone, mangiferin, the flavanone, hesperidin and isosakuranetin were the major flavonoids common to all the species. A more recent study by Joubert et al. [105] showed that mangiferin, isomangiferin and hesperidin are present in all species. There are evidence of species difference and variation in the phenolic composition of *Cyclopia intermedia* and *Cyclopia subternata*. In-depth characterization of fermented extract of *C. intermedia* revealed in addition to common polyphenols, the presence of flavanones (hesperetin, enocitrin, narirutin and naringenin), flavones (luteolin and diosmetin), isoflavones (formononetin, calycosin and wistin), flavanols such as kaempferol glucosides and coumestans (medicagol, flemmichapparin, sophorocoumestan B) [55,106,107] while the compounds isolated from unfermented *Cyclopia subternata* are flavanones (eriocitrin, narirutin), flavones (luteolin, 5-deoxyluteolin, scolymoside), isoflavone (orobol) and flavan-3-ol, epigallocatechin gallate (EGCG), commonly found in green and black tea [108].

Fermentation is also known to reduce the flavonoid content of honeybush, as studies reported a significantly higher total polyphenols and flavonoids in aqueous extract of unfermented honeybush tea compared to the fermented extract of the same lot [48]. Fermentation also impacts on the antioxidant potential of honeybush. It has been reported that unfermented honeybush exhibited greater antioxidant capacity compared to the fermented product when the oxygen radical absorbance capacity (ORAC) assay was carried out and this was attributed to the decrease in xanthone and total polyphenol content [48,105]. Honeybush, like rooibos has a long tradition of medicinal use with anecdotal evidence suggesting its use as an expectorant in chronic catarrh and pulmonary tuberculosis, an appetite enhancer that aids weak digestion, as well as a stimulator of milk production in breast-feeding women [48,109]. Though, not widely reported on compared to rooibos, available evidence indicated that honeybush and its major flavonoids have biological properties that benefit health. Its antioxidant, anti-inflammatory, anti-mutagenic and cancer inhibition properties are well reported [67,110,111,112].

#### Antidiabetic Effects of Honeybush Herbal Tea and Its Major Polyphenols

Numerous in vitro, in vivo animal and human studies support the beneficial effects of honeybush and some of its major polyphenols for the prevention, management and treatment of type 2 diabetes mellitus (Table 3).

Muller et al. [113] were the first to report the antidiabetic effect of honeybush herbal tea when they demonstrated the efficacy of a hot water extract of *Cyclopia intermedia* in ameliorating hyperglycemia using two diabetic rodent models. In the study, an acute hot water extract of fermented *Cyclopia intermedia* at a dose of 50 mg/kg body weight (equaling a dose of 2.90 mg mangiferin) was able to induce a sustained reduction in fasting blood glucose concentration from at least 3 to 6 h in STZ-induced diabetic rats. To confirm the antidiabetic potential of the extract observed in the STZ-induced diabetic rats, the authors followed up with a chronic study in obese insulin resistant rats (OBIR). Inclusion of the hot water extract in the drinking fluid of these rats for 12 weeks improved the α to β-cells ratio, improve glucose tolerance by reducing fasting blood glucose, while also reducing plasma total cholesterol levels in the OBIR rats. Another study from the same group corroborated the antidiabetic potential of honeybush tea in another *Cyclopia* species. Unfermented extract of *Cyclopia maculata* was reported to improve the cell viability of RIN-5F insulinoma cells and showed mitogenic effect in vitro. Furthermore, in the same study, the unfermented extract was reported to improve glucose tolerance in STZ-induced diabetic rats by ameliorating toxic effects on β-cells and reducing fasting blood glucose concentration with a concomitant reduction of total serum triglyceride to within normal physiological range [114]. According to the authors, the results suggested a beneficial effect of the extract on the liver of the rats and a potential ameliorating effect on diabetic complication associated with hyperglycemia and hypertriglyceridemia. Obesity is one of the risk factors for the development of T2DM and agents with anti-obesity effect are suggested to possess antidiabetic potentials. The anti-obesity effect of fermented *Cyclopia maculata* and unfermented *Cyclopia subternata* was demonstrated by Dudhia et al. [115], where it was reported that both extracts inhibit adipogenesis in 3T3-L1 pre-adipocytes without being cytotoxic. The anti-obesity investigations were further extended by the same group when they demonstrated the ability of fermented *Cyclopia maculata* extract to stimulate lipolysis in matured 3T3-L1 adipocytes [116].

The xanthone mangiferin has been reported to be one of the major polyphenols in honeybush herbal tea with very strong antioxidant properties [105]. In vitro and in vivo evidence has shown that mangiferin and extract of mangiferin-containing plants possess antidiabetic potential. An ethanolic extract of the mangiferin-containing plant, *Swertia kouitchensis,* was reported to inhibit the activity of α-amylase and α-glucosidase while also stimulating insulin secretion in NIT-1 cells [129]. In the same study, the extract improved glucose tolerance, alleviate hyperlipidemia and improved antioxidant capacity in STZ-induced diabetic mice. Sellamuthu et al. [130] reported that mangiferin, isolated from *Salacia chinensis,* showed beneficial effects in STZ-diabetic rats by lowering blood glucose level and alleviating altered biochemical parameters. A follow-up study by the same group indicated that the effects may be due to mangiferin-mediated insulin release and regeneration of destroyed pancreatic β-cells [131]. This result was corroborated by two studies which showed that in young (3-month old) and ageing (12-month old) partially pancreatectomized C57BL/6J mice, mangiferin administration at 30 and 60 mg/kg BW showed antidiabetic effect by improving glucose tolerance and glycemia and also facilitated islet regeneration and β-cells proliferation via the up-regulation of cell cycle checkpoint proteins regulators [117,118]. Furthermore, mangiferin was shown to protect against diabetes and dyslipidemia in diabetic, insulin resistant rats by improving insulin sensitivity, modulating lipid profile and reverting adipokine levels to normal [119]. Eight weeks administration of a mangiferin derivative was reported to significantly and dose-dependently reduced plasma glucose and triglyceride level and increased the number of insulin positive β-cells in db/db mice. An investigation into the molecular mechanism of action of the mangiferin derivative indicated that it increased the uptake of glucose in parallel with phosphorylation of AMPK in 3T3-L1 cells. Furthermore, the mangiferin derivative activated AMPK and its downstream target, acetyl-COA carboxylase in the hypothalamus, liver, muscle and adipose tissues of C57BL/6 mice [132].

A more recent work by Min et al. [120], using structure-based virtual ligand screening identified mangiferin as a potential glucokinase activator. In the same study, using an in vitro cell-based assay, mangiferin was shown to effectively improve postprandial blood glucose levels by promoting a remarkable enhancement of glucose consumption in HepG2 and C2C12 cells. These results were further corroborated in vivo when they showed that the administration of mangiferin (200 mg/kg) for 8 weeks improved glucose tolerance, increased serum insulin level and ameliorate histopathological changes in the liver of db/db mice. In the development of diabetic nephropathy, hyperglycemia-induced ROS production is known to perturb several signaling cascades, including those mediated by PKCs, MAPKs, NF-κβ and TGF-1β. In an STZ-induced diabetic rat model, mangiferin ameliorated diabetic nephropathy by reducing oxidative stress and inhibit the expression of PKCs (PKCα, PKCβ and PKCε), MAPKs (p38, JNK and ERK1/2), NF-κβ and TGF-1β [121]. Results from the same study also showed that mangiferin normalized TNFα release and prevented mitochondrial-dependent apoptotic death in the renal tissue of the diabetic rats. Other underlying mechanisms of mangiferin ability to prevent or delay diabetic nephropathy includes suppression of osteopontin production [133], modulating angiotensin II/angiotensin II type I receptor [134], as well as inhibition of the advanced glycation end products/receptor for advanced glycation end products (RAGE) axis [135]. A more recent study gave further insight into the ability of mangiferin to delay the initiation and progression of diabetic nephropathy when it reported that chronic treatment with mangiferin for 12 weeks significantly decreased albuminuria, inhibited glomerular extracellular matrix expansion and restored the expression of the podocyte marker, nephrin. Furthermore, mangiferin protected podocytes by enhancing autophagy by modulating the AMPK-mTOR-ULK signaling pathway [122].

Hesperidin, a major flavanone found in honeybush is reported to possess a myriad of pharmacological properties, including antioxidant, anti-inflammatory, anticarcinogenic, antihyperlipidemic and antidiabetic effects [136]. In an animal model of T2DM, a hesperidin-rich supplementary diet (0.2 g/kg diet) significantly reduced blood glucose level and increased glycogen concentration and glucokinase activity in the liver of C57BL/KsJ-db/db mice [123]. Using a cell culture system representative of a diabetic state, Choi and Kim [137] reported that hesperetin, the aglycone of hesperidin that is also found in honeybush, attenuates the highly reducing sugar-triggered inhibition of osteoblast differentiation by protecting against oxidative stress, suggesting that it may promote bone recovery in diabetic bone diseases. Oral consumption of hesperidin at 10 g/kg diet for four weeks in STZ-induced diabetic rats was reported to exert hypoglycemic effects by altering the activity of glucose-regulating enzymes and normalize lipid profile, as well as adiponectin levels [124]. Other studies have also shown the potential of hesperidin to ameliorate other diabetes related complications. Diabetic neuropathy is the most common microvascular complication affecting almost 50% of diabetic patients. It is characterized by severe and incessant pain and clinically recognized by persistent burning and tingling sensation in legs and feet, inability to detect heat and cold, as well as loss of vibration sensation and pain perception [136]. Hesperidin administration at 50 and 100 mg/kg was reported to alleviate diabetic neuropathy and reversed neuropathic pain by reducing hyperglycemia, hyperlipidemia and advanced glycated hemoglobin. These effects were adduced to hesperidin ability to down-regulate oxido-nitrosative stress and suppress pro-inflammatory cytokine release [136]. Kandemir et al. [125] reported that hesperidin may be useful in reducing the severity of diabetic nephropathy. In the study, hesperidin orally administered at 200 mg/kg/day for four weeks to STZ-induced diabetic rats reduced serum urea and creatinine levels and decreased oxidative stress by reducing MDA and augmenting GSH and antioxidant enzymes in the kidney. Additionally, hesperidin administration reduced the level of transforming growth factor-beta 1(TGF-1β), inhibited the expression of 8-hydroxy-2’-deoxyguanosine (8-OHdG) and ameliorated the morphological abnormalities induced in the kidney of the rats [125]. Hesperidin enhanced the closure of cutaneous wounds created on the dorsal surface of hind paws of STZ-induced diabetic rats by accelerating angiogenesis and vasculogenesis via a mechanisms that involved the up-regulation of mRNA expression of VEGF-c, Ang-1/Tie-2, TGF-β and Smad-2/3 [126]. The study further showed that hesperidin reduced blood glucose, increased serum insulin and alleviated oxido-nitrosative stress in the skin tissue of the rats. Hesperidin administered at 100 and 200 mg/kg body weight to STZ-induced diabetic rats for twelve weeks, protected against retinopathy by alleviating plasma and retina dysfunction through anti-angiogenic, anti-inflammatory and antioxidative effects, while also reducing AGEs accumulation and inhibiting the polyol pathway [138].

Only few clinical trials demonstrating the antidiabetic effect of hesperidin are found in available literatures. A randomized, double-blind, placebo-controlled clinical trial by Eghtesadi et al. [127] showed that hesperidin supplementation decreased plasma total cholesterol and improve glycemic control and insulin resistance in patients with T2DM. While another similar study in which hesperidin was supplemented for six weeks in T2DM patients, indicated that hesperidin improved antioxidant capacity, reduced oxidative DNA damage and lipid peroxidation but showed no effect on fasting blood glucose and insulin resistance [128]. Other evidence in animal model of T2DM has shown that other honeybush flavonoids such as naringenin, narirutin and isosakuranetin showed antidiabetic potentials via different molecular mechanisms. Details of these studies have been reviewed by Alam et al. [139] and Barreca et al. [140].

## 3. Conclusions and Future Perspectives

In conclusion, this review has shown that the two South African herbal tisanes—rooibos and honeybush are rich sources of natural bioactive compounds, with unquestionable evidence of potent antidiabetic properties from in vitro, in vivo and few available human studies. Therefore, their consumption might reduce the risk of T2DM and its complications. However, the molecular mechanisms of how these herbal tisanes and their bioactive components impact on the major endpoints of T2DM are still inconclusive. This may be due to the complex and variable risk factors of diabetes and likely cofounding effects of lifestyle and background dietary influence. Therefore, more well-designed epidemiologic and prospective human studies are still needed to evaluate the relevance of rooibos and honeybush in the prevention, management and treatment of T2DM.

## Figures and Tables

**Table 1:** Some phytochemical constituents of *Camellia sinensis* tea, rooibos and honeybush herbal teas.

*Camellia sinensis*	Rooibos (*Aspalathus linearis*)	Honeybush (*Cyclopia* species.)
Caffeine	Aspalathin	Hesperetin
Gallic acid	Nothofagin	Hesperidin
Gallocatechin	Iso-orientin	Isosakuranetin
Catechin	Orientin	Naringenin
Epicatechin	Isovitexin	Eriodictyol
Epigallocatechin gallate	Vitexin	Eriocitrin
Gallocatechin	Chrysoeriol	Luteolin
Gallocatechin gallate	Luteolin-7-*O*-glucopyranoside	Kaempherol
Epicatechin gallate	Quercetin-3-*O*-rubinoside	Medicagol
Rutin	Hyperoside	Narirutin
	Rutin	Mangiferin
	Isoquercitrin	Isomangiferin
	Phenylpyruvic acid-2-*O*-glucopyranoside	Formononetin
	Eriodictyol	Calycosin
	Caffeic acid	Wistin
	Ferulic acid	Fujikinetin
	p-Coumaric acid	Epigallocatechin gallate

**Table 2:** Summary of Studies Indicating Potential anti-T2DM Potential of Rooibos and/or Rooibos Polyphenols.

Test Material/Dose/Route of Administration	Duration	Described Effects and Mechanisms	Reference(s)
Aqueous- (5 mL/kg BW) or alkaline-extract (300 mg/kg BW) of fermented rooibos administered to STZ-induced diabetic rats.	8 weeks	Diabetic status not altered, but extract lower levels of oxidative stress markers including lipid peroxidation, malondialdehyde and AGEs in the plasma, lens and liver	[69]
Fermented rooibos extract (2%), as sole source of drinking fluid administered to rats after induction of diabetes (STZ, 50 mg/kg body weight)	7 weeks	Inhibition of STZ-induced oxidative stress via suppression of lipid peroxidation and enhancing the activity SOD and glutathione peroxidase	[70]
Cardiomyocytes isolated from STZ-induced diabetes rats pre-treated with fermented rooibos extract (1 µg/mL or 10 µg/mL), before exposure to H_2_O_2_ or an ischemic solution	6 hours	Fermented rooibos extract protected against cardiomyocytes apoptosis, cardiomyocytes intracellular ROS generation and GSH content depletion induced by H_2_O_2_ (1 µg/mL) or by ischemia (1 µg/mL and 10 µg/mL)	[71]
11-day old L6 myotubes cultured in buffer containing 11 mM glucose with or without aspalathin (0–100 µM)	4 hours	Aspalathin dose-dependently increased glucose uptake at concentration 1–100 µM with maximum stimulatory effect at 10 µM	[73]
RIN-5F cells derived from pancreatic β-cells exposed to RPMI medium containing 0–100 µM aspalathin	3 hours	Aspalathin at 100 µM stimulated insulin secretion from cultured RIN-5F cells	
0.1% or 0.2% aspalathin supplemented in the diet of db/db mice	5 weeks	Aspalathin (0.1% and 0.2% in diet) suppressed the fasting blood glucose level and alleviate impaired glucose tolerance in db/db mice	
C2C12 myotubules exposed to aspalathin-enriched green (unfermented) rooibos (5 × 10^−5^ to 50 µg/mL), pure aspalathin and rutin (1 × 10^−4^ to 100 µM)	1 hour	Aspalathin-enriched green rooibos promotes glucose uptake by C2C12 myotubules at all concentrations tested. The increased glucose uptake was comparable to metformin (a hypoglycemic agent) at concentration of 0.05–5 µg/mL. Pure aspalathin and rutin showed increased glucose uptake at 1–100 µM and 100 µM respectively.	[74]
Aspalathin-enriched green rooibos extract (2.5 to 50 mg/kg BW), pure aspalathin, rutin and a 1:1 mixture of aspalathin-rutin combination (1.44 mg/kg BW) administered orally to STZ-induced diabetic rats	6 hour	The aspalathin-enriched green rooibos induced a sustained reduction in plasma glucose concentration over a 6 hour period with maximum effect at 5 and 5 mg/kg BW. Extract also showed a glucose lowering effect comparable to that of metformin at 25 mg/kg BW. Pure aspalathin and aspalathin-rutin mixture reduced the blood glucose concentration of the diabetic rats at 6 and 4 hour respectively after oral administration.	
Aspalathin-enriched green rooibos extract (3, 30 or 300 mg/kg BW) administered orally to STZ-induced diabetic rats, followed by oral administration of glucose (2 g/kg BW) an hour later	4 hours	Extract at 30 mg/kg BW decreased glucose concentration at 60, 120 and 240 min by 27.3, 33.7 and 58%. Increasing the concentration of extract to 300 mg/kg BW did not improve reduction of glucose concentration	
Mice orally administered glucose, maltose, sucrose, or starch (0.2 g/mL/100g BW), with or without green (unfermented) rooibos extract (40 mg/mL) and aspalathin (8 mg/mL)	3 hours	Aspalathin-enhanced green rooibos suppressed the elevation of blood glucose after glucose, maltose and starch overload at 30 and 60 min after administration. Aspalathin showed suppressive effects on blood glucose elevation at 30 min (glucose and maltose overload), at 30, 60, 90 and 120 min (sucrose overload) and at 60 min (starch overload).	[75]
Mice orally administered aspalathin-enriched green rooibos extract (80 mg/mL/100 g BW) or pure aspalathin (20 mg/mL/100 g BW), and 2 hour later received intraperitoneal injection of glucose (0.2 g/mL/100 g BW)	2 hours	Both aspalathin-enriched green rooibos and pure aspalathin did not show any suppressive effect on blood glucose level using the intraperitoneal glucose tolerance test (IPGTT).	
Aspalathin-enriched green rooibos or pure aspalathin on in vitro α-glucosidase and α-amylase inhibitory activity	Up to 1 hour	Both aspalathin-enriched green rooibos extract and pure aspalathin showed dose-dependent inhibition of α-glucosidase (maltase and sucrase) and α-amylase activities	
Palmitate-induced insulin resistant C2C12 cells treated with aspalathin-enriched green (unfermented) rooibos extract or a hot water extract of fermented rooibos	3 hours	Both aspalathin-enriched green rooibos and fermented rooibos extract increased glucose uptake, mitochondrial activity and ATP production in both basal and insulin-stimulated palmitate-treated C2C12 cells. Aspalathin-enriched green rooibos more effective at increasing glucose uptake and ATP production Both rooibos extracts down-regulated *PKCθ* activation and up-regulated AKT and AMPK which are both key regulatory proteins involved in insulin-dependent and non-insulin regulatory pathways. Protein levels of glucose transporter GLUT4 involved in glucose transport via the two pathways were increased by both rooibos extracts increasing glucose uptake	[76]
Palmitate-induced insulin resistant 3T3-L1 adipocytes treated with aspalathin-enriched green (unfermented) rooibos (GRE, 10 µg/mL) or pure aspalathin (ASP, 10 µM)	3 hours	Both GRE and ASP increased glucose uptake, mitochondrial activity and ATP production in basal and insulin-stimulated palmitate-treated 3T3–L1 adipocytes GRE (basal and insulin-stimulated) and ASP (basal) increased fatty acid uptake Both GRE and ASP suppressed NF-κβ protein expression, reduced insulin receptor substrate one (serine 307) (IRS1 (ser^307^)) and AMPK phosphorylation, and increased AKT expression GRE alone increased GLUT4 expression while ASP alone increased the protein expression of PPARα, PPARγ and carnitine palmitoyltransferase 1 (CPT1)	[77]
L6 myotubes and RIN-5F pancreatic β-cells treated with ASP (0–100 µM)	4 hours	ASP dose-dependently increased glucose uptake, promoted AMPK phosphorylation and enhanced GLUT4 translocation to plasma membrane in L6 myotubes. In RIN-5F cells, ASP reduced oxidative stress by suppressing AGE-induced rise in ROS	[78]
Obese diabetic ob/ob mice fed with diet containing ASP (0.1%)	5 weeks	ASP suppressed increase in fasting plasma glucose levels, and decreased expression of hepatic genes related to gluconeogenesis and lipogenesis	
L6 myotubes and RIN-5F pancreatic β-cells treated with GRE (0–800 µg/mL) or ASP (50 µM)	4 hours	GRE increased glucose uptake in the absence of insulin, induced phosphorylation of AMPK and AKT, and enhanced translocation of GLUT4 to plasma membrane in L6 myotubes. GRE protect cultured RIN-5F cells from oxidative stress by suppressing AGE-induced increase in ROS.	[79]
Diabetic KK-A^y^ fed a diet containing GRE at 0.3% (first 3 weeks) or 0.6% (following 2 weeks)	5 weeks	GRE reduced increase in fasting blood glucose levels	
HUVECs subjected to high-glucose (HG)-induced inflammation treated with ASP or nothofagin (NOT) (0–50 µM)	6 hours	Both ASP and NOT prevented high-glucose-mediated vascular hyperpermeability, adhesion of monocytes, and expression of cell adhesion molecules. Both compounds inhibited generation of ROS and activation of NF-κβ and ERKs.	[80]
Mice subjected to HG-induced inflammation treated with ASP (4.5, 9.1, 27.1 and 45.2 µg/mouse) or NOT (4.4, 8.7, 26.2, 43.6 µg/mouse) intravenously	6 hours	Both ASP and NOT inhibited HG-induced vascular permeability in vivo.	
High-Fat fed diabetic Vervet monkeys administered GRE (90 mg/kg BW, three times daily)	4 weeks	GRE improved glucose tolerance, protected against LDL oxidation, preserved endogenous coenzyme Q10 levels and decrease oxidative stress.	[81]
LPS-activated HUVECs treated with ASP or NOT	6 hours	Both ASP and NOT inhibited LPS-induced barrier disruption, expression of CAMs, and adhesion/transendothelial migration of neutrophils to human endothelial cells. Both compounds suppressed production of TNF-α or IL-6 and activation of NF-κβ ERK 1/2 induced by LPS.	[82]
LPS-stimulated mice administered ASP (9.1, 18.2 and 27.1 µg/mouse) or NOT (8.7, 17.4 and 26.2 µg/mouse) intravenously	6 hours	Both ASP and NOT suppressed LPS-induced hyper-permeability and leucocyte migration, and reduced LPS-induced lethal endotoxemia.	
HUVECs subjected to high-glucose (HG)-induced inflammation treated with orientin or isoorientin (0–50 µM)	6 hours	Orientin and iso-orientin dose-dependently decreased HG-mediated endothelial cell membrane disruption and adhesion of THP-1 cells to HUVECs. Both compounds showed maximum inhibition of CAMs expression at 50 µM.	[84]
Mice subjected to HG-induced inflammation administered orientin or isoorientin (9, 18, and 45 µg/mouse)	6 hours	Both orientin and iso-orientin protected against HG-mediated vascular permeability.	
High-fructose (HF)-fed obese mice administered isoorientin (20 or 40 mg/kg BW)	8 weeks	Iso-orientin improved lipid profiles and alleviate HF-induced lipid metabolic disorders. Iso-orientin alleviated HF-induced oxidative liver injury by reducing lipid peroxidation, enhancing antioxidant enzymes activities and ameliorating histopathological changes in the liver. Iso-orientin prevented HF-induced inflammation in the liver by inhibiting increase in levels of TNF-α, IL-1 and IL-6.	[86]
Vitexin or Isovitexin orally administered to sucrose loaded normoglycemic mice (1, 3, and 15 mg/kg BW) and STZ-induced diabetic rats	90 min	Vitexin dose-dependently reduced postprandial blood glucose level at 30 min. Isovitexin at all doses used reduced postprandial blood glucose level at 30 min. However, only 3 and 15 mg/kg BW reduced postprandial blood glucose level at 60 min.	[88]
STZ-induced diabetic rats administered orally with vitexin (50, 100 and 200 mg/kg BW) or isovitexin (20, 50 and 100 mg/kg BW)	90 min	Vitexin (200 mg/kg BW) and isovitexin (100 mg/kg BW) reduced postprandial blood glucose level in diabetes-induced rats.	
Vitexin and isovitexin inhibition of AGEs formation in an in vitro bovine serum albumin (BSA)-glucose model	7 days	Both compounds inhibited formation of AGEs induced by glucose or methylglyoxal with efficacy of 85% at 100 µM.	[89]
Diet-induced obese mice fed luteolin (0.005%) in diet	16 weeks	Luteolin lowered fasting blood glucose, improved glucose tolerance and modulated the activities of hepatic glucose-regulating enzymes. It lowered plasma insulin concentration and improved hepatic insulin sensitivity by suppressing the expression of SREBP1.	[91]
STZ-induced diabetic rats treated with luteolin (50 and 100 mg/kg BW)	8 weeks	Treatment with luteolin improved cognitive dysfunction, significantly ameliorated cholinergic dysfunction and attenuated oxidative stress in diabetic rats.	[92]
STZ-induced diabetic rats administered with luteolin (200 mg/kg BW) via an intragastric tube	8 weeks	Luteolin reversed histopathological dysfunction in the kidney, reduced blood glucose and returned the values of serum kidney function markers to near normal, improved serum and kidney lipid profile and alleviated kidney oxidative stress.	[93]
STZ-induced diabetic rats administered with rutin (50 mg/kg BW) as an intraperitoneal injection once a week	45 days	Rutin reduced blood glucose and improved lipid profile. Rutin also restored the changes observed in the activities of ALT, AST and LDH in the serum, liver and heart of diabetic rats.	[94]
STZ-induced diabetic rats administered orally with rutin (100 mg/kg BW)	45 days	Rutin decreased fasting plasma glucose and increased plasma insulin level. Diabetes induced changes in liver, kidney and muscle glycogen content and activities of hexokinase, glucose-6-phosphatse and fructose-1,6-biphosphatase were restored. Rutin also protected against diabetes-induced oxidative stress by reducing lipid peroxidation and augmenting antioxidant enzymes activities.	[96]
STZ-induced diabetic mice administered PPAG (10 mg/kg BW)	11 days	PPAAG delayed the onset of hyperglycemia and prevented β-cells mass destruction induced by STZ, by inhibiting β-cells apoptosis	[98]
INS-1E cells pre-incubated with PPAG (30 µM) for 16 hour and exposed to STZ (1 mM)	17 hours	PPAG reduced STZ-induced cell death by about 30–40%	
Human islets exposed to 0.5 mM palmitate with or without PPAG (30 µM)	3 days	PPAG also protected β cells from human islets against lipotoxic injury induced by palmitate	
High fat- and fructose fed obese mice orally administered PPAG (10 mg/kg BW)	6 weeks	PPAG protected against increase in fasting plasma glucose and insulin level. B-cell mass was increased by suppressing apoptosis of β-cells	[99]
Palmitate-treated INS-1E and β-cells isolated from human islet exposed to PPAG (0–100 µM and 30 µM respectively )	16 or 24 hours	PPAG protected INS-1E (dose-dependently) and human β-cells (30 µM) against palmitate and other ER stress-induced apoptosis by restoring the expression of BCL2 protein	
Chang (CCL-13) cells exposed to log and semilog dilutions of PPAG (100–0.001 µM)	3 hours	PPAG stimulated significant increases in glucose uptake by Chang cells within the concentration range 1.0–31.6 µM	[100]
Insulin-deficient STZ-induced diabetic rats orally administered PPAG (0.14 and 1.4 mg/kg BW)	6 hours	Acute administration of PPAG has no effect on 16-hour fasted blood glucose level of diabetic rats over a 6-hour period	
Obese insulin-resistant (OBIR) orally administered PPAG either as a single dose (0.1, 3 and 10 mg/kg BW) or a 0.3 mg/kg BW per day (2 weeks) and an increase to 3 mg/kg BW per day (1 week)	3 weeks	3 mg/kg BW dose reduced 16-hour fasted blood glucose level over a 6 hour period. Lower daily dose has no effect on 16-hour fasting blood glucose concentration after 2 weeks, but increased dose of 3 mg/kg BW reduced 16-hour fasted blood glucose concentration.	
High-glucose exposed H9C2 cardiomyocytes treated with PPAG (1 µM)	48 hours	PPAG protected against hyperglycemia-induced substrate impairment, mitochondrial depolarization and cell apoptosis	[101]
Normolipidemic individuals administered fermented rooibos after intake of standardized fat meal	6 hours	Fermented rooibos modulated postprandial glycemia, lipemia and oxidative stress in subjects	[102]

**Table 3:** Summary of Some Studies Indicating Potential anti-T2DM Potential of Honeybush and/or Honeybush Polyphenols.

Test Material/Dose/Route of administration	Duration	Described Effects and Mechanisms	Reference(s)
STZ-induced diabetic rats orally administered single dose (0, 5, 25, 50 mg/kg BW) of honeybush extract (HBE)	6 hours	HBE at a dose of 50 mg/kg BW reduced the fasting blood glucose concentration of diabetic rats at 4, 5 and 6 hours	[113]
OBIR orally administered HBE (538, 1075, 1792, 2150 or 2688 mg/100 mL) in drinking fluid	12 weeks	HBE at all doses used reduced the hyperglycemic fasting blood glucose and total plasma cholesterol level in OBIR rats. HBE (1075, 1792, 2150 or 2688 mg/100 mL) reduced the in vitro glucose tolerance test (IVGTT) values after 12 weeks of treatment. HBE (538, 1075, 1792, 2150 mg/100 mL) reduced the α-cell mass and increased the α-cell to β-cell ratio	
RIN-5F cells exposed to STZ (10 mM) and treated with *Cyclopia maculata* extract (0.01–2000 µg/mL)	24 hours	*Cyclopia maculata* extract improved cell viability of RIN-5F insulinoma cells and showed no mitogenic effect *in vitro*	[114]
Rats orally administered *Cyclopia maculata* extract (30 or 300 mg/kg daily) for 15 days, then injected intra-peritoneally with STZ and further treated with extract for another 6 days	21 days	*Cyclopia maculata* extract improved glucose tolerance and total serum tryglyceride level in the diabetic rats. Also the extract increased β-cell area to total islet area, as well as β-cell proliferation in the diabetic rats	
3T3-L1 mouse adipocytes exposed to *Cyclopia maculata* (fermented and unfermented) and *Cyclopia subternata* (unfermented) extract (0–1600 µg/mL) in adipogenesis inducing and maintenance medium	8 days	All extracts used inhibited intracellular tryglyceride and fat accumulation, and also decreased PPARγ2 expression	[115]
Differentiated 3T3-L1 adipocytes exposed to *Cyclopia maculata* (fermented and unfermented) and *Cyclopia subternata* (unfermented) extract (0–100 µg/mL)	24 hours	Fermented *Cyclopia maculata* extract at 80 µg/mL induced maximal lipolysis measured as glycerol concentration in culture supernatant. This is accompanied by increased protein expression of hormone sensitive lipase and perilipin	[116]
3-month-old partially pancreatectomized mice administered mangiferin (30 or 90 mg/kg BW)	14 days	Mangiferin exhibited improved glycemia and glucose tolerance, increased serum insulin levels, enhanced β-cell hyperplasia, elevated β-cell proliferation and reduced β-cell apoptosis. Critical genes related to β-cell regeneration (PDX-1, Ngn3, GLUT2, Foxo-1 and GCK), as well as key regulators of cell cycle (cyclin D1, D2 and Cdk4) were upregulated at either/or both mRNA and protein expression level	[117]
Aged (12-month-old) partially pancreatectomized mice administered mangiferin (90 mg/kg BW)	28 days	Mangiferin decreased blood glucose and increased glucose tolerance accompanied by increased serum insulin level. Treated mice exhibited islet hyperplasia, elevated β-cell proliferation and reduced β-cell apoptosis. The expression of cyclin D1, D2 and Cdk4 were upregulated while that of p16^INK4a^ and p27^Kip1^ were downregulated at both mRNA and protein level	[118]
Diabetic insulin-resistant rats administered mangiferin (20 mg/kg BW, i.p.)	28 days	Mangiferin administration reduced serum glucose level, improved insulin resistance and increased β-cell function. Serum and hepatic lipid profile was improved and level of TNF-α and adiponectin in the serum was increased	[119]
HepG2 and C2C12 myotubes incubated with various concentration (0–1 mM) of compound identified as mangiferin by structure-based virtual ligand screening	24 hours	Mangiferin induced a remarkable dose-dependent and a relative enhancement of glucose consumption in HepG2 and C2C12 myotubes respectively	[120]
db/db mice administered mangiferin (200 mg/kg BW)	8 weeks	Mangiferin improved glucose tolerance and increased serum insulin level. Histopathological changes induced in diabetics mice was reversed by mangiferin	
STZ-induced diabetic rats administered mangiferin (40 mg/kg BW) orally	30 days	Mangiferin lowered ROS production and decreased intracellular antioxidant defenses. It also ameliorated diabetic nephropathy (DN) by modulating the MAPK (p38, JNK and ERK1/2), PKC isoforms (PKCα, PKCβ and PKCε), TGF-1β pathways and NF-κβ signaling cascades	[121]
STZ-diabetic rats administered mangiferin (12.5, 25 and 50 mg/kg BW)	12 weeks	Mangiferin decreased albuminuria, restored the expression of nephrin, a podocyte marker and inhibited glomerular extracellular matrix expansion. Thus showing ability to delay the initiation and progression of DN	[122]
db/db mice supplemented with hesperidin (0.2 g/kg diet)		Hesperidin reduced blood glucose level, and increased glycogen concentration, as well as hepatic glucokinase activity	[123]
STZ-induced marginal type 1 diabetic rats administered hesperidin (10 g/kg diet)	4 weeks	Hesperidin decreased blood glucose by altering the activity of glucokinase, and normalized lipid profile in the rats	[124]
STZ-induced diabetic rats administered hesperidin (200 mg/kg BW)	4 weeks	Hesperidin reduced serum urea and creatinine levels. In the kidney, levels of MDA, TGF-1β and 8-OHdG were reduced while glutathione concentration was increased	[125]
Cutaneous-wounded, STZ-induced diabetic Sparague-Dawley (SD) rats administered hesperidin (25, 50, and 100 mg/kg)	21 days	Hesperidin reduced blood glucose, increased serum insulin and alleviated oxido-nitrosative stress in the skin tissue of the rats. Hesperidin enhanced closure of cutaneous wound via mechanisms involving up-regulation of mRNA expression of VEGF-c, Ang-1/Tie-2, TGF-β and Smad-2/3	[126]
T2DM patients supplemented with hesperidin (500 mg/day)	8 weeks	Hesperidin decreased fasting blood glucose, glycated hemoglobin and total cholesterol while increasing the level of serum insulin	[127]
T2DM patients supplemented with hesperidin (500 mg/day) in a randomized, double-blind, placebo-controlled clinical trial	6 weeks	Hesperidin improved antioxidant capacity, reduced oxidative DNA damage and lipid peroxidation, but showed no effect on fasting blood glucose and insulin resistance	[128]
